# Habit a Factor to Be Considered in the Treatment of Regulation Cases

**Published:** 1899-09

**Authors:** George Brunton

**Affiliations:** Leeds


### Habit a Factor to be considered in the Treatment of Regulation Cases.

BY GEORGE BRUNTON, LEEDS.

Read at the Midland Counties Branch Meeting, held at Blackpool, February 25, 1899.

The particular bad habit to which I would draw your attention is pushing the mandible out of its normal position, and pressing the lower against the upper teeth in such a way that crowding and irregularity are induced. The models of a case which I hand round will illustrate what I mean. No. 1 is the normal bite, No. 2 shows the bad habit of biting forward which has produced malposition in at least ten teeth. This case is a boy of 14 years. Three years ago I corrected the position of the upper left central incisor, which stood inside the arch. No retaining plate was made as it was not considered necessary, the lower teeth locking the incisor forward. Unfortunately the boy was not brought for examination until a few days ago, when the mother's attention was drawn to the irregular position and the habit of biting forward. She said that the *habit* had been noticed but *not* the irregularity. The advice given was "Cure the boy of the bad habit and *then* I will undertake the regulation of the teeth." Treatment on any other conditions I thought would be without any good result.—*Journ. of the British Dental Association.*

A NUMBER of the best known local dentists have organized a movement to perfect an Academy of Dental Science in San Francisco, on the lines on which other institutions of a similar character are being successfully conducted in other parts of America. The objects of the Association are to advance the interests of dentistry, and to encourage closer fraternal relations among the members. It is proposed to meet each month at some well-known hotel or restaurant, and after a banquet, papers on topics germane to the profession will be read. It is believed that the organization will be perfected before the close of the present month.

